# Identification of gully erosion activity and its influencing factors: A case study of the Sunshui River Basin

**DOI:** 10.1371/journal.pone.0309672

**Published:** 2024-11-21

**Authors:** Fengjie Fan, Xingli Gu, Jun Luo, Bin Zhang, Hui Liu, Haiqing Yang, Lei Wang

**Affiliations:** 1 School of Geographical Sciences, China West Normal University, Nanchong, China; 2 Sichuan Provincial Engineering Laboratory of Monitoring and Control for Soil Erosion in Dry Valleys, China West Normal University, Nanchong, China; 3 Liangshan Soil Erosion and Ecological Restoration in Dry Valleys Observation and Research Station, Xide, China; 4 College of Geography and Environmental Sciences, Zhejiang Normal University, Jinhua, China; ICIMOD: International Centre for Integrated Mountain Development, NEPAL

## Abstract

Gully erosion is one of the most severe forms of land degradation and poses a serious threat to regional food security, biodiversity, and human survival. However, there are few methods for the quantitative evaluation of gully activity, and the relationships between gully activity and influencing factors require further in-depth study. This study takes the Sunshui River Basin, as a case study. Based on field investigation, unmanned aerial vehicle (UAV) photography and remote sensing images, 71 typical gullies were identified. The vegetation coverage (VC), slope and main-branch gully ratio (MBGR) were used as evaluation indicators, and the gully activity was calculated using the fuzzy mathematics membership degree and then evaluated quantitatively. The factors influencing different active gullies were also analyzed. The results showed that (1) the fuzzy comprehensive evaluation method can be used to identify gully activity. Different levels of gully activity were defined based on the gully activity index. The active indices of stable gullies ranged from 0–0.25, those of semiactive gullies ranged from 0.25–0.75, and those of active gullies ranged from 0.75–1. (2) The activity indices of the 71 gullies ranged from 0.054 to 0.999, with an average value of 0.656. There are 31 active gullies, and 31 semiactive gullies. A total of 87.32% of the gullies in the study area were in the early or middle stage of gully development. Gully erosion was intense, which is consistent with the serious reality of soil erosion. (3) Gully activity was affected by multiple factors. It was significantly positively correlated with topographic relief (TR) (r = 0.64, *P*<0.01) and surface curvature (SC) (r = 0.51, *P*<0.01), while it was significantly negatively correlated with land use type (LUT) (r = -0.5, *P*<0.01). Surface roughness (SR) (r = 0.2, *P*<0.01) was positively correlated with gully activity; but not significantly. There was no significant correlation between aspect (As) and gully activity. The results of this study are helpful for quantitatively determining the level of gully activity and understanding the development process and mechanism controlling gullies, providing a reference for research on related regions and geomorphologic information.

## Introduction

Soil erosion is among the major environmental problems facing humanity, posing serious threats to regional food security, biodiversity and the human living environment, and severely restricting regional sustainable development [1]. Gully erosion is one of the most important forms of soil erosion, resulting in the loss of approximately 5×10^5^−7×10^5^ hectares of arable land each year [2]. Gully erosion primarily occurs in semiarid and arid regions [3], and gullies are the main source of river sediment, transporting 70% of all sediment [4, 5]. Moreover, the occurrence and development of gully erosion aggravates water and soil loss problems, reduces land resources and productivity, and significantly impacts agricultural production. Furthermore, gully erosion poses a serious threat to the safety of downstream rivers and lakes, increasing the frequency of downstream flood disasters and worsening the ecological environment [6, 7].

Gully erosion is a major surface runoff manifestation, concentrated by the erosion of surface soil and parent material and the formation of gullies via cutting into the ground surface. Triggered by multiple factors, such as surface vegetation [8], soil [5, 9], meteorological factors [10], human activities [11], and topography [12, 13]. VC is crucial in determining gully erosion’s evolution stage [14]. It can directly affect the gully erosion rate [15], while vegetation roots can indirectly reduce the erosion rate by improving soil cohesion and tensile strength of the soil-root matrix [16, 17]. Soil mechanical composition impacts its resistance to erosion [18, 19], but high soluble mineral content increases susceptibility to infiltration and leakage, affecting soil shrinkage, cohesion, and particle structure. Reduced soil organic matter stability promotes crust formation, runoff, and erosion [20, 21]. Precipitation influences each erosion stage differently. Catchment area precipitation provides water and potential energy for runoff, which contributes to gully formation and evolution [22, 23]. Long-term rainfall or concentrated infiltration causes soil water saturation, promoting initial erosional cut formation in both small and large gullies [24], while continuous runoff leads to sidewall scouring and collapse, increasing gully depth and releasing sediments that are subsequently transported out of the gully. Additional erosion and small gully erosion related to direct rainfall occur on gully sidewalls [25]. If bedrock, water tables or impermeable layers are reached, gully erosion intensifies [26, 27]. The runoff process in the catchment area is related to the rainfall amount, rainfall intensity, and antecedent rainfall, while large-scale destruction processes within the gully system (such as gully collapse) are more related to the overall soil saturation [28]. At the same runoff volume, steeper slopes lead to faster runoff velocities and larger runoff volumes in gullies, and steeper slopes are more conducive to the development and propagation of gullies than gentle slopes [29]. With increasing rainfall intensity, slope, and catchment area, the soil loss increases. The topographic relief and runoff volume strongly impact gully erosion in downstream areas [30, 31].

As the research scope of gully erosion shifts from individual gullies to entire catchment areas, more focus has been placed on the quantitative evaluation and evolution of gully erosion [32]. However, due to differences in surface characteristics among regions, there is currently no unified quantitative standard for determining the evolution stage of gully erosion. The existing related research has involved mainly the development of gully erosion [2], gully classification, morphological characteristics, erosion risk evaluation, gully activity and influencing factors [33]. The determination of gully activity has involved mainly qualitative methods and semiquantitative models. Qualitative methods are used to evaluate gully activity through field observations and interpretation of aerial photos [34, 35]; however, these methods rely on professional knowledge and are somewhat subjective. With the application of advanced technologies, such as high-precision GPS [36, 37], 3D laser scanners [38, 39], aerial photos and photogrammetry [40], for studying gully characteristics [41, 42], semiquantitative models, such as factor scoring models (FSMs) and analytic hierarchy process (AHP) [43, 44], principal component analysis (PCA) [45–47], and geomorphic information entropy, have gradually been applied in soil science and gully erosion studies. However, the 3D laser scanner accurately scans the gully but requires considerable time to scan an entire gully. Satellite remote sensing images can extract and study the terrain parameters of large areas but have relatively low accuracy, leading to large errors. Meanwhile, quantitative research on gully activity mostly focuses on determining gully head activity, with few quantitative descriptions of activity across entire gullies [48]. In the existing studies on the division of different gully erosion, gully activity is categorized as active, semiactive and stable [49, 50]. Current research on gullies mainly uses GPS, 3D laser scanning and satellite remote sensing images to study the occurrence mechanism, distribution and erosion amount estimation of gullies, or to monitor short-term dynamic changes on a small spatial scale [51]. However, there is a lack of research on judging the overall gully characteristics and activity of the gully area by using high-resolution UAV remote sensing images.

The fuzzy comprehensive evaluation method, based on the theory of membership degree in fuzzy mathematics, transforms qualitative evaluations into quantitative ones [52, 53]. It has been applied to studies on the evaluation of debris flow gully activity and the assessment of river ecological stability [54]. However, there have been no reports on the evaluation of gully activity. The activity of gullies is a complex and variable natural phenomenon, and its activity level is often difficult to describe in precise mathematical language. Fuzzy mathematics provides an effective tool to quantify this ambiguity. Therefore, this study applies the fuzzy comprehensive evaluation method to evaluate gully activity, aiming to: a) calculate the activity index of each gully and classify the gully activity level; b) explore the influencing factors of gully activity. The research results will help understand the natural process of gully erosion, improve the ability to predict and warn of geological disasters, and provide a scientific basis for local soil erosion prevention, efficient water and soil resource utilization, and sustainable soil and water conservation measures implementation.

## Materials and methods

### Study area

**Fig 1:**
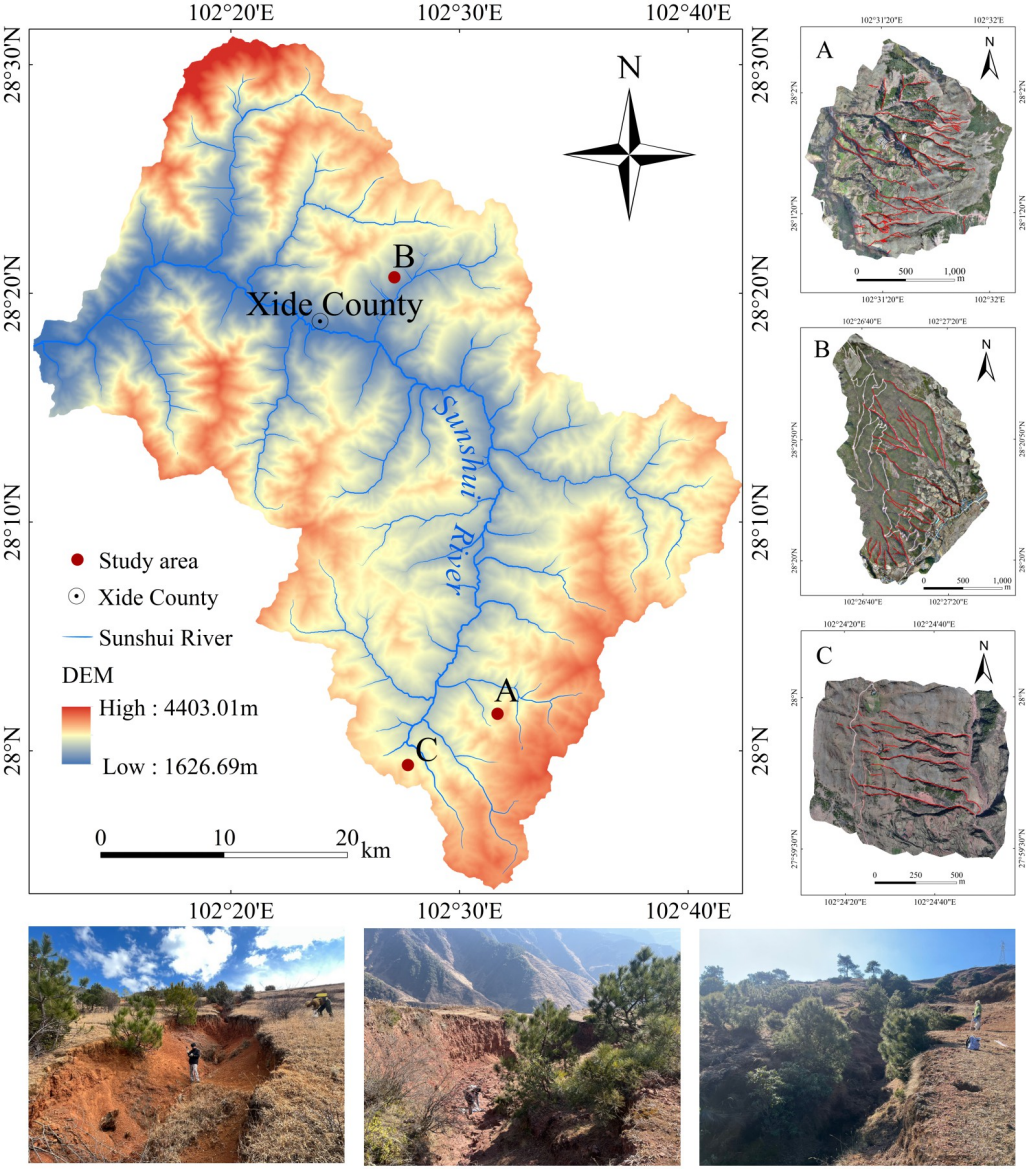
Study area. Republished from<Identification and influencing factors of gully activity in the Sunshui river basin> under a CC BY license, with permission from China West Normal University, original copyright 2003.

The Sunshui River is a tributary of the left bank of the midstream region of the Anning River in the Jinsha River system, with a total length of 95.2 km and a catchment area of 1617.5 km^2^. Most sections of the Sunshui River are in Xide County, which has a subtropical monsoon climate, an average temperature of 14–17°C and an average rainfall of approximately 1100 mm. The Sunshui River has a natural flow of 1424 m, an average flow rate of 40.3 m^3^/s, and a runoff volume of 1.197 ×10^8^ m^3^ [55]. The soil type is purple soil. The forest vegetation in the Sunshui River Basin has been severely damaged, leading to severe soil erosion. Eighty percent of the sediment in the Yalong River Basin originates from the Anning River Basin, and 80% of the sediment in the Anning River Basin comes from the Sunshui River. Large amounts of sediment increase flood disasters in the Anning River Basin. If the Sunshui River is not controlled, the Anning River will not be stable. This study selected three typical areas in the Sunshui River Basin as the key research areas (A (28°02′N, 102°32′E), B (28°20′N, 102°26′E), and C (28°00′N, 102°28′E)) (Fig 1). The terrain in the study area is typical and uniform. The terrain is higher in the east and lower in the west. The eastern side comprises mainly mountainous terrain, while the western side is mainly river valley plain terrain. The altitude ranges from 1927.37 m to 3070.76 m, and there are significant differences in the development of gully erosion. These gullies basically represent different stages of gully development in the basin.

### Data acquisition and processing

The research site is located in Xide County, Liangshan Yi Autonomous Prefecture, which is mainly engaged in agriculture and animal husbandry. The research site in this paper is a completely open and free exploration area, and no permits were required.

#### Data sources

**Fig 2:**
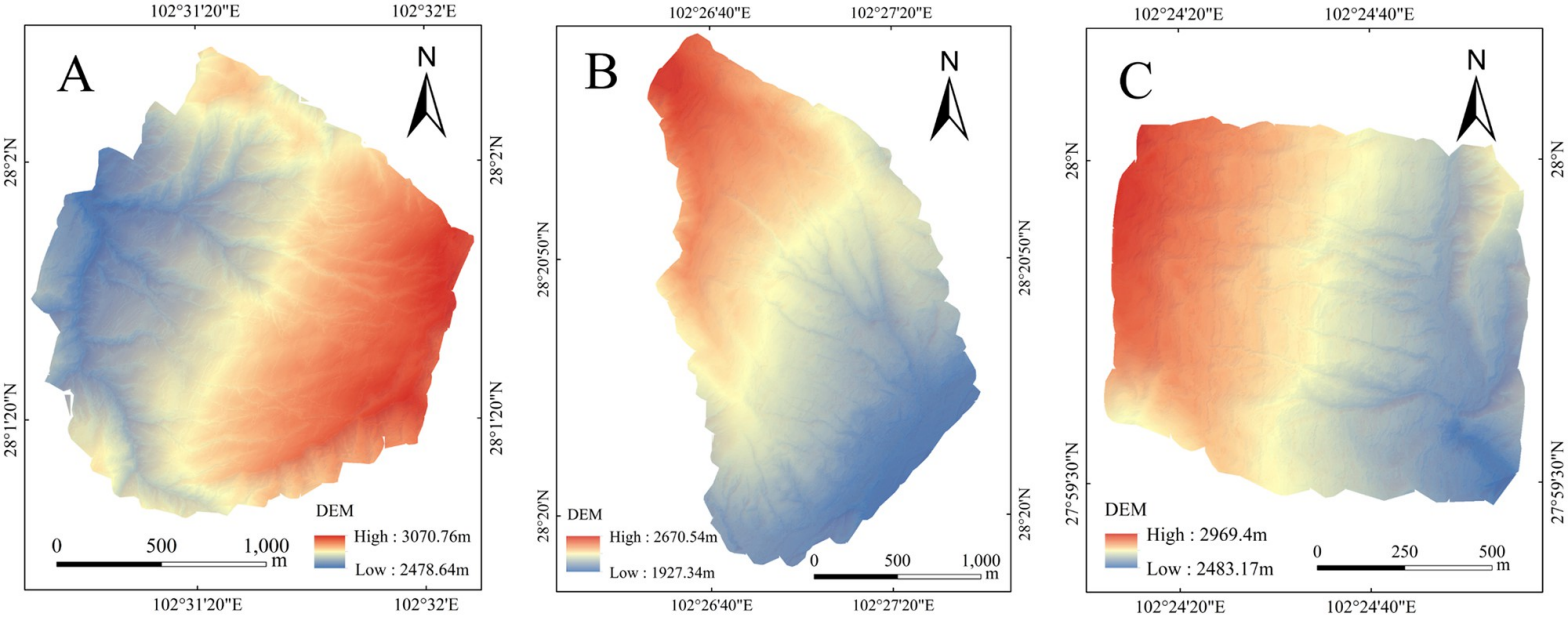
The processed DEM map of the study area.

In this study, UAV photography technology and a Feima D200 UAV (Shenzhen Pegasus robot Co., LTD, Shenzhen, China) flight platform equipped with a LiDAR laser lens were used to collect data. The main technical steps included image control point layout and measurement. The image control point layout was established according to the requirements of the "Airborne Photogrammetry Field Specification for Topographic Maps of 1:500, 1:1000, and 1:2000 (GB/T 7931)" and the "Low-Altitude Digital Aerial Photogrammetry Field Specification (CH/Z 3004–2010)." Real-time kinematic (RTK) data were used to collect image control points for accuracy verification. Line planning and camera parameter settings accounted for the flight attitude, flight angle, and shooting frequency of the aircraft. With respect to the camera parameters, the heading overlap was 45%, the point density was 49 points/m^2^, the speed was 8 m/s, the altitude was 70 m, the flight altitude was changed, and the coordinate system was WGS 1984. The airborne POS data and LiDAR raw data were processed using UAV management software to output classified point cloud data and a DEM with a resolution of 0.5 m (Fig 2).

#### Data acquisition method

In the region of severe soil erosion at the headwater of the Sunshui River, gullies are widely developed and concentrated. In this study, 71 gullies with different vegetation types, land use types and development levels were selected as research subjects to identify gully erosion activity and its influencing factors.

**Fig 3:**
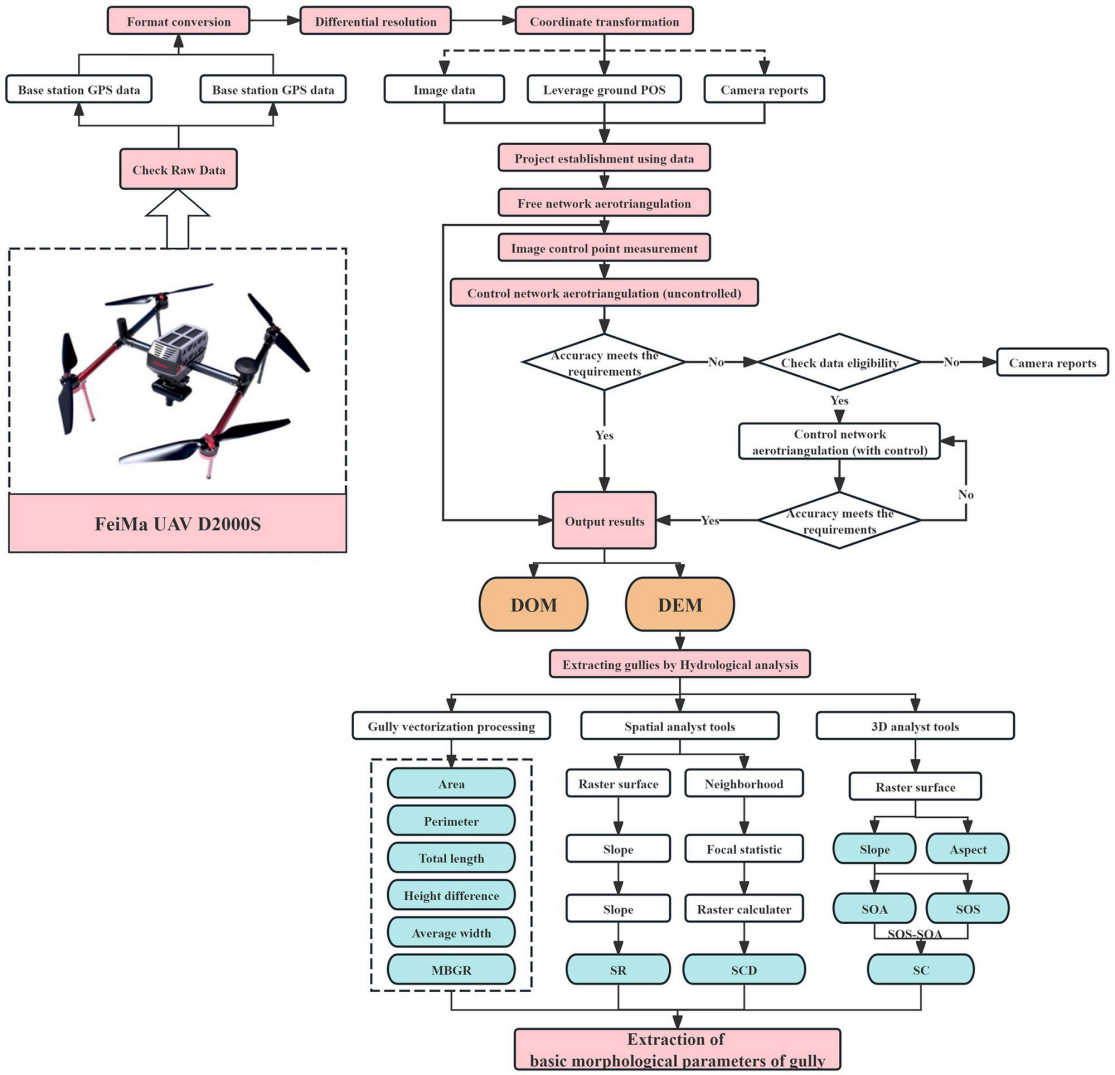
Data acquisition process. The filling color block unit is the basic morphological parameter.

**Fig 4:**
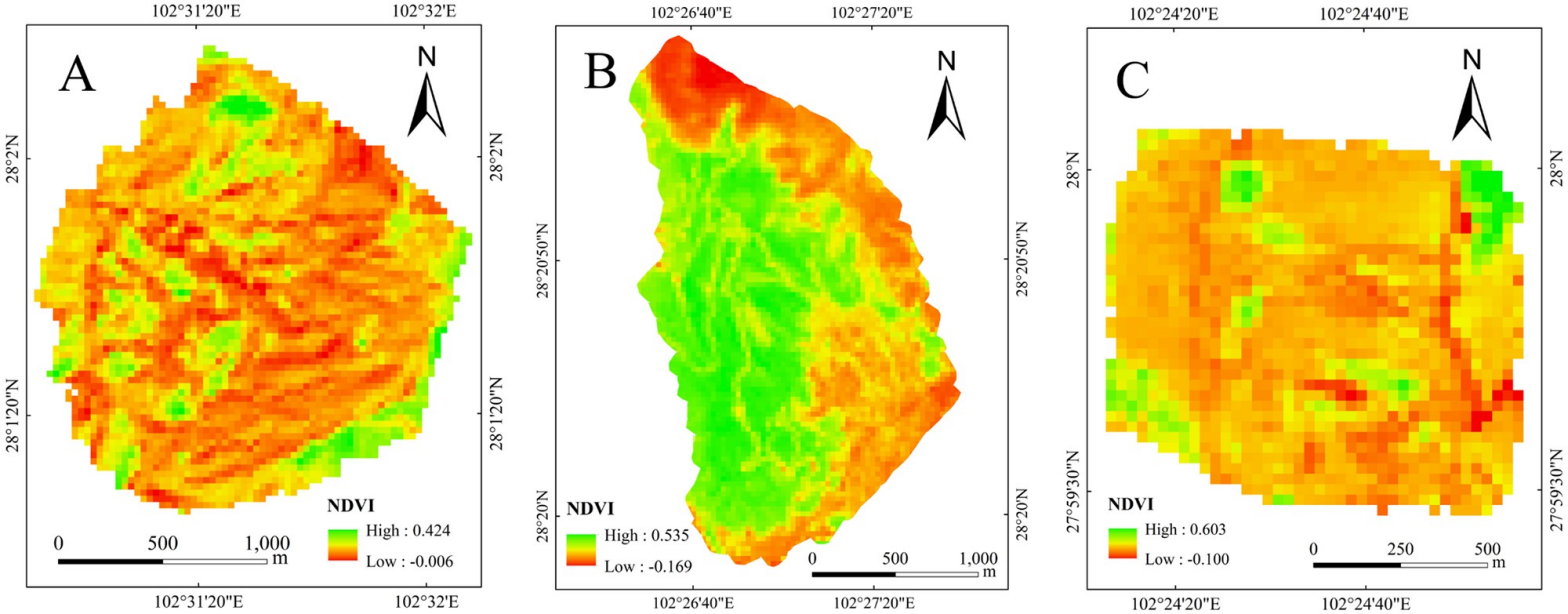
NDVI map of study area.

(1) Basic morphological parameters: Using ArcGIS (10.7) software, gullies were extracted through hydrological analysis to obtain data, including the gully erosion area, circumference, and total gully length. Using ArcGIS’s 3D Analyst, Spatial Analyst, and other modules, a digital elevation model (DEM) of the study area was used as the data source to extract gully slope, aspect, surface cutting depth (SCD), topographic relief (TR), and surface curvature (SC) data, and statistical analysis was performed on the basic characteristic data. The data acquisition process is shown in Fig 3.

(2) VC: The data source is the Landsat 8 OLI_TIRS high-resolution remote sensing image from November 2021. The normalized difference vegetation index (NDVI) was calculated using ENVI 5.6 software to indicate the VC in the gully, reflecting the stability level and erosion capacity of the gully erosion (Fig 4).

Vegetation index (VI) is a numerical value obtained by linear or nonlinear combination of infrared band (R) and near-infrared band (IR) in multi-spectral remote sensing data through mathematical operations, which can be used to characterize the vegetation cover and quality of the surface. Normalized Vegetation Index (NDVI) is commonly used, and the formula is as follows(1).

$$\text{NDVI}=\left(\text{NIR}-\text{R}\right)/\left(\text{NIR}+\text{R}\right)$$ (1)

Where NIR is the near-infrared band and R is the infrared band. There is a significant correlation between VC and normalized Vegetation index (NDVI). Generally, the correlation between VC and normalized vegetation index (NDVI) is used to estimate the VC of the study area by referring to the research of Hou Yong et al. on grassland in Inner Mongolia. The calculation formula is as follows (2).

$$\text{VC}={\text{NDVI-NDVI}}_{\text{min}}/{\text{NDVI}}_{\text{max}}-{\text{NDVI}}_{\text{min}}$$ (2)

Where VC is the vegetation coverage, NDVI_min_ is the bare soil coverage pixel in the study area, NDVI_max_ is the pure vegetation coverage pixel in the study area, and the calculated FVC vegetation coverage value should vary between 0 and 1.

**Fig 5:**
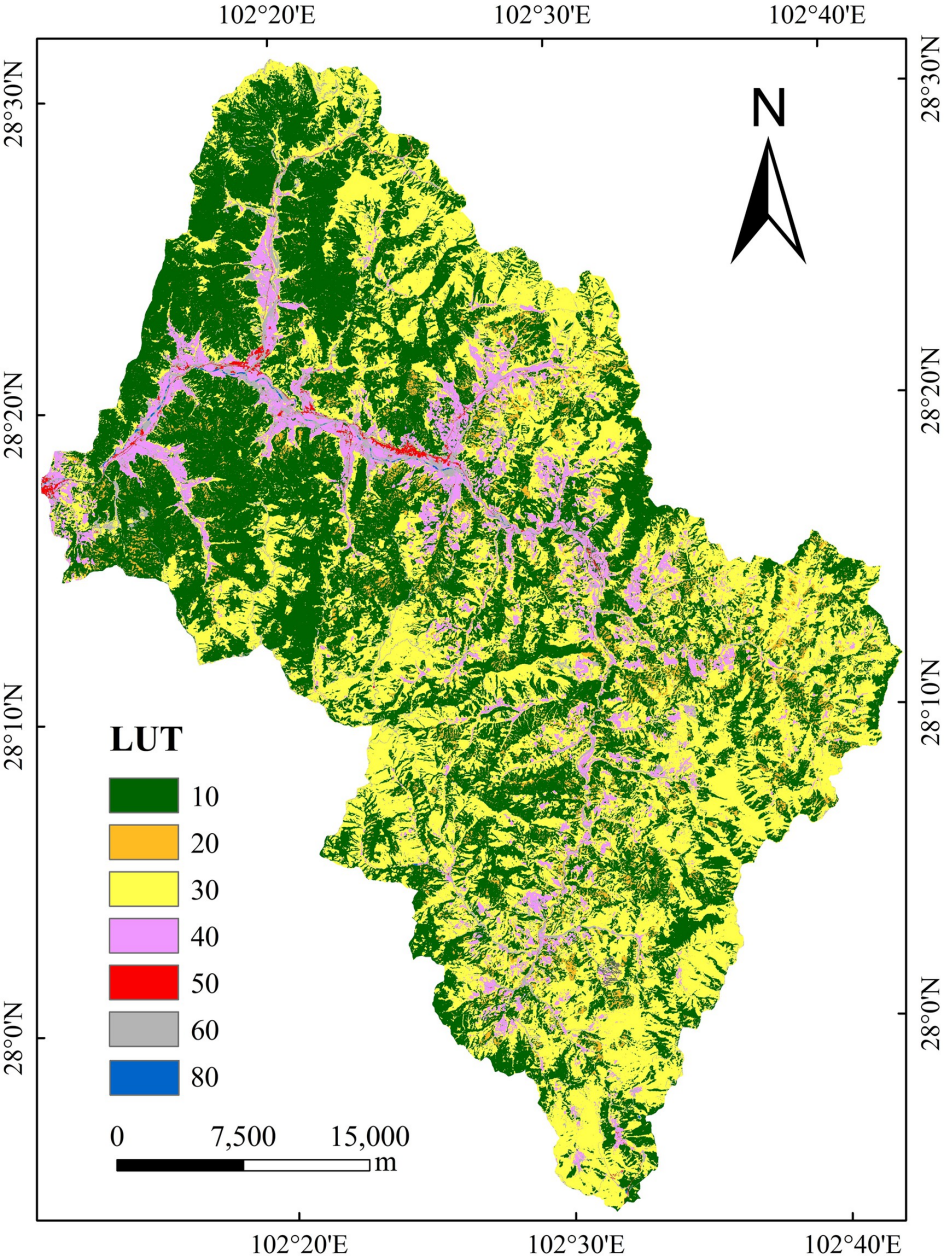
Land use type map of Sunshui River basin.

(3) Land use type (LUT): The LUT data come from the 2021 land cover dataset, which has a 10-metre accuracy and was jointly produced and released by the European Space Agency (ESA) in collaboration with several scientific research institutions worldwide (Fig 5). The data were generated based on the data obtained from Sentinel-1 and Sentinel-2 in 2020, with the geographic coordinate system being GCS_WGS_1984, and the projection type was the UTM projection. LUTs are defined by the land cover classification system developed by the Food and Agriculture Organization of the United Nations (FAO), as shown in Table 1.

**Table 1:** Land use classification.

Code	Land use type	RGB
10	Forestland	0,100,0
20	Shrub land	255,187,34
30	Grassland	255,255,76
40	Cultivated land	240,150,255
50	Building land	250,0,0
60	Bare land	180,180,180
70	Snow, ice	240,240,240
80	Water body	0,100,200
90	Wetland	0,150,160
100	Mangrove	0,207,117
110	Moss, lichen	250,230,160

#### Research methods

This study primarily quantifies the activity level of gullies based on the membership degree of fuzzy mathematics. Membership degree is a fundamental concept in fuzzy mathematics, used to describe the degree to which an element belongs to a fuzzy set. In a fuzzy set, the degree of belonging to the set is represented by a value between 0 and 1, known as the membership degree [56]. The calculation steps are as follows:

(1) Deterministic fuzzy set

Based on the different development levels of gully erosion and the actual conditions of the study area, gully activity was divided into three levels to establish a fuzzy set, namely, *V = {V*_*1*_, *V*_*2*_, *V*_*3*_*}* = {active, semiactive, stable}, to characterize the evaluation set.

(2) Select evaluation index

Comprehensive assessment of gully activity requires selection of a series of indicators that can quantitatively reflect varying levels of erosion. A thorough review of literature on gully erosion development and influencing factors reveals that VC, slope, and MBGR are pivotal indicators that either affect or directly reflect the activity level of gully erosion. Therefore, this study utilizes these three indicators to comprehensively assess the erosion characteristics of gullies. If there is a factor set *U = {U*_*1*_,*U*_*2*_,*U*_*3*_,*…*,*U*_*m*_*}* consisting of m factors that affect the evaluation object and an evaluation set *V = {V*_*1*_,*V*_*2*_,*V*_*3*_,*…*,*V*_*m*_*}* consisting of n evaluation grades, then the active relationship between gully erosion activity and evaluation factors can be represented by a fuzzy evaluation matrix *R* as follows (3):

$$R=r\left(i,j\right),i=1,2,\ldots ,m;j=1,2,\ldots ,n$$ (3)

where *R* represents the fuzzy relationship between factor set *U* and evaluation set *V* and *r(i*,*j)* is the membership degree of the *i*th factor in factor set *U* to the *j*th evaluation level in evaluation set *V*.

(3) Determine the membership function.

For each evaluation index, a membership function must be specified to convert the index’s specific value into the corresponding membership degree. This study employs a commonly used trapezoidal function (Table 2) to calculate membership degrees. The selection of a membership function typically depends on expert experience and measured data. The membership functions of VC, slope and MBGR are determined as follows:

**Table 2:** Trapezoidal function.

Interval	Activity level		
	Active	Semiactive	Stable
*x* ≤ *a*	1	0	0
*a* < *x* < *b*	$\frac{b-x}{b-a}$	$\frac{x-a}{b-a}$	0
*b* < *x* < *c*	0	$\frac{c-x}{c-b}$	$\frac{x-b}{c-b}$
*x* ≥ *c*	0	0	1

In the table, the threshold value between the active and semiactive types is A, and the threshold value between the semiactive and stable types is B. Then, a = A, b = (A+B)/2, and c = B.

1) VC

VC is a key factor in determining the evolution stage of gully erosion, and the gully erosion decreased with increasing vegetation [15]. Due to differences in natural environments, there is currently no unified quantitative standard for evaluating gully erosion stages using VC. The overall VC in the study area is low, When VC is less than 10%, the gully vegetation is low cover and close to bare land, and the gully is active. Under slow shear at the gully bottom, the gully wall begins to widen, and there are some deposits at the bottom of the gully, which can support current plant growth, but the vegetation is sparse, with coverage generally between 10%-30%, indicating medium-low coverage, and the gully is a semi-active erosion trench. As cutting down of the gully essentially stops, the longitudinal profile of the gully bottom is close to the equilibrium profile, the gully bottom topography is relatively open, there are more sediments at the gully bottom, and a large number of plants grow at the gully bottom and wall, with shrubs, small trees, or artificial forests present, and the VC is greater than 30% [57, 58]. At this time, the gully belongs to stable gully. This is the basis for dividing gully activity (Table 3).

**Table 3:** Subordinate function of gully activity under different VC(%).

Interval	Activity level		
	Active	Semiactive	Stable
*x* ≤ 10	1	0	0
10 < *x* < 20	$\frac{20-x}{20-10}$	$\frac{x-10}{20-10}$	0
20 < *x* < 30	0	$\frac{30-x}{30-20}$	$\frac{x-20}{30-20}$
*x* ≥ 30	0	0	1

2) Slope

The slope is another influential factor in gully erosion [3]. Different slopes exert varying forces on surface runoff after precipitation, affecting soil aggregation and leading to gully erosion [59]. The slope is significantly correlated with soil moisture content, water retention capacity, and water demand. Slope is the primary terrain factor affecting the number of shallow gullies on slopes [2]. Meanwhile, other scholars have concluded that soil erosion is controlled by soil properties, precipitation, slope, topography, and slope length, among other factors, with slope having the most significant influence [48, 60]. Therefore, slope can be used as an index to judge the degree of soil erosion. The headwater area of the Sunshui River also belongs to the alpine and gorge region, and geomorphic types with slopes less than 25° are rare. When slopes are large, various primary gullies will collapse and landslide under the action of gravity, no longer suitable for gully formation and development, and soil erosion also shifts from gully erosion to gravity erosion. Based on the actual situation in the study area, this article defines gullies with a slope of ≥35° as active, 25°–35° as semiactive, and ≤ 25° as stable (Table 4).

**Table 4:** Subordinate function of gully activity under different slopes.

Interval	Activity level		
	Active	Semiactive	Stable
*x* ≤ 25	0	0	1
25 < *x* < 30	0	$\frac{x-25}{30-25}$	$\frac{30-x}{30-25}$
30 < *x* < 35	$\frac{x-30}{35-30}$	$\frac{35-x}{35-30}$	0
*x* ≥ 35	1	0	0

3) MBGR

The MBGR of a gully reflects the development degree and quantity of the branch gullies in the gully system from the perspective of the length of the gully and indirectly reflects the degree of fragmentation of the gully system [61]. The smaller the MBGR is, the more significant the development of branch gullies in the gully system, and the greater the development degree of the gully system. Zhao Jiaying et al. studied the characteristics of gully erosion in the Nanxiaohe River Basin and classified gullies into three types based on the MBGR: main gully type, semi-main gully type, and tributary gully type [62]. Based on the actual conditions of the study area, this article defines gullies with main and branch gully ratios ≤0.5 as active gullies, 0.5–1 as semiactive gullies, and ≥1 as stable gullies (Table 5).

$$R=\frac{L_{0}}{L}$$ (4)

Where R is MBGR of a gully, L_0_ main gully length; L is the total length of the valley.

**Table 5:** Classification of gully activity under different MBGR.

Interval	Activity level		
	Active	Semiactive	Stable
*x* ≤ 0.5	1	0	0
0.5 < *x* < 0.75	$\frac{0.75-x}{0.75-0.5}$	$\frac{x-0.5}{0.75-0.5}$	0
0.75 < *x* < 1	0	$\frac{1-x}{1-0.75}$	$\frac{x-0.75}{1-0.75}$
*x* ≥ 1	0	0	1

(4) Comprehensive evaluation of gully activity
1) Determination of evaluation factor weights

Let the set of weight factors *A = {a*_*1*_,*a*_*2*_,*a*_*3*_*…a*_*m*_*}* be used to evaluate each object, where a_1_ is the weight corresponding to the *ith* evaluation factor and $\sum_{i=1}^{m}a_{i}=1$. This study used principal component analysis (PCA) to determine the weight of each evaluation factor. PCA is a multivariate statistical analysis method that can divide multiple related indicators into a few independent comprehensive indicators. In many semiquantitative models in soil science, PCA has been widely used to determine the weight of indicators.

2) Establish fuzzy matrix

The obtained evaluation factor weight set *A* is subjected to fuzzy transformation with the fuzzy evaluation matrix *R* to obtain the comprehensive evaluation set *B*, which characterizes the degree of membership in the activity level. In the formula, *b*_*J*_ is the degree of membership of the evaluation factor to the Jth activity level. According to the principle of maximum membership, *maxb*_*J*_ is the gully erosion activity degree.


$$B=A\cdot R=\left(\text{b}1,\text{b}2,\ldots \text{bn}\right)=\left(\text{a}1,\text{a}2,\ldots \text{am}\right)\cdot \left[\begin{array}{c} \begin{array}{ccc} r_{11}r_{12} & \ldots & r_{1n}\\ \;r_{21}\;\;\;\;r_{22} & \ldots & r_{2n}\\ \vdots \;\;\;\;\;\;\;\vdots & \cdots & \vdots \end{array}\\ r_{m1}\;\;\;r_{m2}\;\;\;\cdots \;\;\;r_{mn} \end{array}\right]$$ (5)

3) Calculation and division of gully activity

In the above model, we obtained a comprehensive evaluation result B of gully activity, but this comprehensive evaluation result has a disadvantage of discontinuity, for example, it is impossible to distinguish which gully is more active in the same level. This defect can be solved by using a fuzzy comprehensive index [54, 63]. According to the common classification of gully activity in the arid valley area of Southwest China, the gully activity is divided into three levels, and the construction vector H = (0, 0.5, 1) is used to assign values to three grades. The larger the value, the more active the gully is, and the calculation formula is as follows.

$$Q=B\times H=\sum_{1}^{n}B_{i}H_{i}$$ (6)

where H is the evaluation set status point, that is, {stable, semiactive, active} = {h = 0, h = 0.5, h = 1}, and B is the fuzzy comprehensive evaluation set.

Since the evaluation results are generally not integers, when the activity value is near a certain evaluation setpoint, the activity corresponding to the evaluation setpoint can be used as the evaluation result. For instance, when the activity index of an gully is 0.29, it is closest to 0.5 among the three state points (0, 0.5, 1), corresponding to the semiactive state. Thus, the gully is defined as semiactive gully. That is, the stable gully activity index is 0–0.25, the semiactive gully activity index is 0.25–0.75, and the active gully activity index is 0.75–1.

## Results and analysis

### Gully activity index calculation

Using principal component analysis (PCA) to calculate the weights of the three indicators, the weights of VC, slope, and MBGR are 0.361, 0.282 and 0.357, respectively. The actual values of the evaluation factors for the 71 gullies in the study area are entered into the corresponding membership functions, and the membership degrees of the evaluation factors for different activity levels can be obtained. Writing them in matrix form gives the fuzzy evaluation matrix.


$$\begin{array}{l} {\text{R}}_{\text{A}01}=\left[\begin{array}{ccc} 0.22 & 0.78 & 0\\ 1 & 0 & 0\\ 0 & 0 & 1 \end{array}\right]\;{\text{R}}_{\text{A}02}=\left[\begin{array}{ccc} 0.71 & 0.39 & 0\\ 1 & 0 & 0\\ 0.72 & 0.28 & 0 \end{array}\right]\;{\text{R}}_{\text{A}03}=\left[\begin{array}{ccc} 0.32 & 0.68 & 0\\ 1 & 0 & 0\\ 0 & 0 & 1 \end{array}\right]\\ {\text{R}}_{\text{A}04}=\left[\begin{array}{ccc} 0.86 & 0.14 & 0\\ 1 & 0 & 0\\ 0.32 & 0.68 & 0 \end{array}\right]\;{\text{R}}_{\text{A}05}=\left[\begin{array}{ccc} 0.96 & 0.04 & 0\\ 1 & 0 & 0\\ 0 & 0 & 1 \end{array}\right]\;{\text{R}}_{\text{A}06}=\left[\begin{array}{ccc} 0.91 & 0.09 & 0\\ 0.02 & 0.98 & 0\\ 0 & 0 & 1 \end{array}\right]\\ {\text{R}}_{\text{A}07}=\left[\begin{array}{ccc} 0.97 & 0.03 & 0\\ 1 & 0 & 0\\ 0.72 & 0.28 & 0 \end{array}\right]\;{\text{R}}_{\text{A}08}=\left[\begin{array}{ccc} 0.23 & 0.77 & 0\\ 0 & 0 & 1\\ 0 & 0 & 1 \end{array}\right]\;{\text{R}}_{\text{A}09}=\left[\begin{array}{ccc} 1 & 0 & 0\\ 0 & 0.1 & 0.9\\ 0.64 & 0.36 & 0 \end{array}\right]\\ \ldots \ldots \end{array}$$

The weight set A is multiplied by the fuzzy evaluation matrix R of the 71 gullies in the study area, resulting in a comprehensive evaluation set of activities for the 71 gullies.


$$\begin{array}{c} B_{1}=A\cdot R_{A01}=\left\{0.354,0.289,0.357\right\};\\ B_{2}=A\cdot R_{A02}=\left\{0.792,0.208,0\right\};\\ B_{3}=A\cdot R_{A03}=\left\{0.390,0.253,.357\right\};\\ B_{4}=A\cdot R_{A04}=\left\{0.721,0.279,0\right\};\\ \vdots \\ B_{n}=A\cdot R=\left({\text{b}}_{1},{\text{b}}_{2},\ldots {\text{b}}_{\text{n}}\right)=\left({\text{a}}_{1},{\text{a}}_{2},\ldots {\text{a}}_{\text{m}}\right)\cdot \left[\begin{array}{cccc} r_{11} & r_{12} & \ldots & r_{1n}\\ r_{21} & r_{22} & \ldots & r_{2n}\\ \vdots & \vdots & \ldots & \vdots \\ r_{m1} & r_{m2} & \ldots & r_{mn} \end{array}\right] \end{array}$$

The calculation results of activity index of 71 gullies in the study area (Table 6) show that there are 31 active gullies, 31 semiactive gullies, and 9 stable gullies.

**Table 6:** Activity index of 71 gullies in the study area.

Number	AI	Level	Number	AI	Level
A01	0.499	semiactive	A37	0.900	active
A02	0.896	active	A38	0.481	semiactive
A03	0.517	semiactive	A39	0.845	active
A04	0.861	active	B01	0.838	active
A05	0.643	semiactive	B02	0.910	active
A06	0.487	semiactive	B03	0.946	active
A07	0.950	active	B04	0.817	active
A08	0.217	stable	B05	0.932	active
A09	0.668	semiactive	B06	0.729	semiactive
A10	0.718	semiactive	B07	0.928	active
A11	0.464	semiactive	B08	0.982	active
A12	0.249	stable	B09	0.433	semiactive
A13	0.204	stable	B10	0.778	active
A14	0.533	semiactive	B11	0.999	active
A15	0.235	stable	B12	0.946	active
A16	0.963	active	B13	0.964	active
A17	0.279	semiactive	B14	0.964	active
A18	0.644	semiactive	B15	0.999	active
A19	0.735	semiactive	B16	0.874	active
A20	0.752	active	C01	0.849	active
A21	0.876	active	C02	0.643	semiactive
A22	0.795	active	C03	0.574	semiactive
A23	0.350	semiactive	C04	0.643	semiactive
A24	0.365	semiactive	C05	0.535	semiactive
A25	0.635	semiactive	C06	0.419	semiactive
A26	0.250	stable	C07	0.805	active
A27	0.571	semiactive	C08	0.464	semiactive
A28	0.739	semiactive	C09	0.546	semiactive
A29	0.814	active	C10	0.235	stable
A30	0,999	active	C11	0.893	active
A31	0.940	active	C12	0.199	stable
A32	0.777	active	C13	0.553	semiactive
A33	0.746	semiactive	C14	0.822	active
A34	0.463	semiactive	C15	0.054	stable
A35	0.714	semiactive	C16	0.181	stable
A36	0.456	semiactive			

### Verification of calculation results

**Fig 6:**
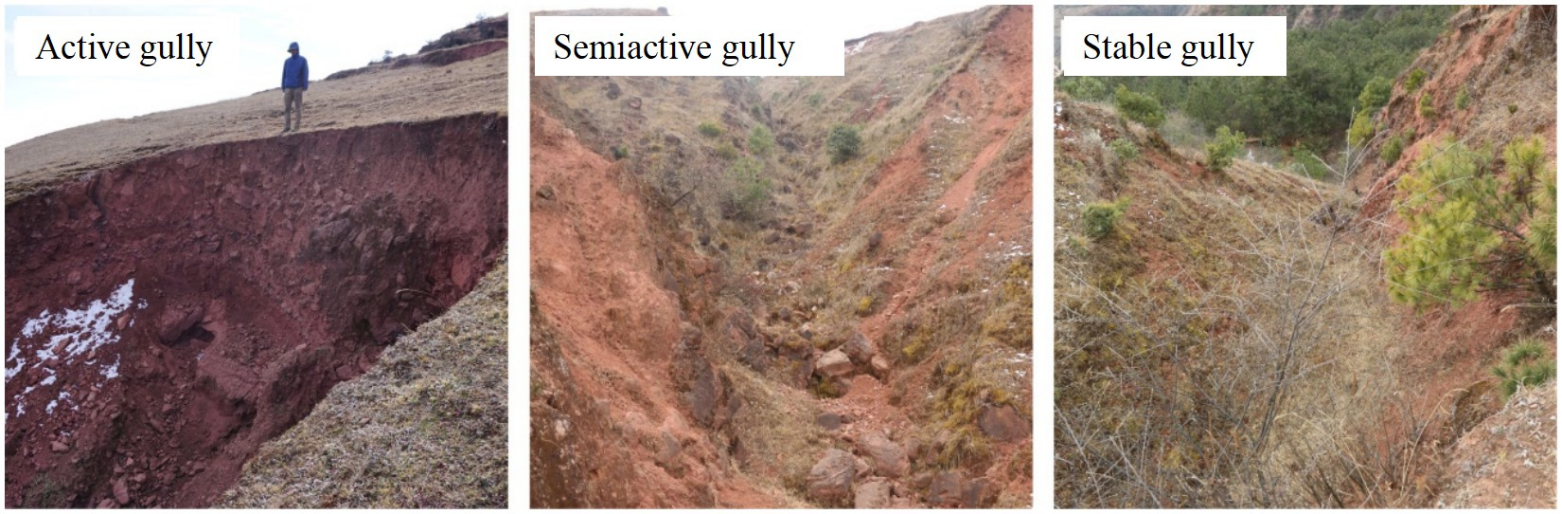
Schematic representation of the level of gully activity.

The judgment of gully activity is mainly based on empirical judgment and semi-quantitative model calculation. In this study, the activity values of 71 gullies are calculated by fuzzy comprehensive evaluation model, and the results are verified by empirical judgment method. According to the empirical judgment, the activity of gullies is classified according to the activity of gully heads and VC. Among them, the active gullies are exposed on the ground, with low VC(C<10%), and all of them are mainly herbaceous layers, with developed gully heads and steep slope of gully walls, which are prone to collapse, common fresh colluvium, and accompanied by severe traceability erosion. The surface of semi-active gully is exposed, the VC is gradually increased(10%<C< 30%), the community structure of herb layer and shrub layer is relatively perfect, the diversity index is relatively high, the gully head falls low, the slope of the gully wall is relatively slow, and fresh colluvium is occasionally seen, so the traceability erosion speed of the gully head is slow or stopped; The VC of the gully bed in the stable gully is relatively high (30% < C), and the slope of the gully wall is relatively slow. Under the combined action of water power and gravity, the original gully surface basically does not collapse, and the gully head stops the source erosion (Fig 6).

Based on the field investigation of the gullies, gully head erosion and VC, combined with the interpretation of DOM data in the study area, 71 gullies in the study area are divided into three types: active, semiactive and stable, and the division results are compared with the quantitative calculation results (Table 7). There are 66 gullies with consistent results, accounting for 92.96% of the total, and 5 gullies with inconsistent results, accounting for 7.04% of the total. The five gullies with inconsistent results are divided into active gullies in empirical judgment, but they are divided into semiactive gullies in fuzzy comprehensive evaluation method, and their activity indexes are all between 0.7 and 0.75, which are very close to the critical value of active gullies. Therefore, the model can accurately distinguish the gully with relatively stable erosion from the gully with relatively active erosion, but it cannot accurately (< 100%) distinguish the active gully from the semiactive gully. There is a transitional relationship between active gully and semiactive gully. In the empirical judgment, besides the established factors, the physical crust at the gully head, tensile cracks, loose deposits at the bottom of the gully and other factors are also considered, while the fuzzy comprehensive evaluation method only inputs the established factors, so there may be errors in the definition of active and semiactive gullies. However, the advantage of this model is that it can accurately calculate the activity index of each gully, and it can accurately judge which gully is more active or stable under the same type of gully conditions, which is impossible for empirical judgment.

**Table 7:** Empirical judgment results and comparison.

BH	EJ result	QD result	BH	EJ result	QD result	BH	EJ result	QD result
A01	2	2	A25	2	2	B10	3	3
A02	3	3	A26	1	1	B11	3	3
A03	2	2	A27	2	2	B12	3	3
A04	3	3	A28	2	2	B13	3	3
A05	2	2	A29	3	3	B14	3	3
A06	2	2	A30	3	3	B15	3	3
A07	3	3	A31	3	3	B16	3	3
A08	1	1	A32	3	3	C01	3	3
A09	3	2	A33	3	2	C02	2	2
A10	3	2	A34	2	2	C03	2	2
A11	2	2	A35	3	2	C04	2	2
A12	1	1	A36	2	2	C05	2	2
A13	1	1	A37	3	3	C06	2	2
A14	2	2	A38	2	2	C07	3	3
A15	1	1	A39	3	3	C08	2	2
A16	3	3	B01	3	3	C09	2	2
A17	2	2	B02	3	3	C10	1	1
A18	2	2	B03	3	3	C11	3	3
A19	3	2	B04	3	3	C12	1	1
A20	3	3	B05	3	3	C13	2	2
A21	3	3	B06	3	2	C14	3	3
A22	3	3	B07	3	3	C15	1	1
A23	2	2	B08	3	3	C16	1	1
A24	2	2	B09	2	2			

In the table, EJ result is the result of empirical judgment and QD result is the result of quantitative division. 1 stands for stable gully, 2 stands for semi- active gully and 3 stands for active gully.

### Morphological characteristics of gullies with different levels of activity

In the table, EJ result is the result of empirical judgment and QD result is the result of quantitative division. 1 stands for stable gully, 2 stands for semi- active gully and 3 stands for active gully.The morphological characteristics and parameters of gullies are specific manifestations of their characteristics at different levels of activity. Therefore, this article describes the characteristics of gullies at different levels of activity based on 6 types of morphological characteristics and parameters, namely, the gully area, perimeter, total length, height difference, average width and surface cutting depth. The overall scale of gully erosion in the study area is not large. There are 30 gullies with an area ≤1000 m^2^, accounting for 42.25% of the total; 38 gullies with a perimeter ≤500 m account for 47.89% of the total; 53.52% of the gullies have a length ≤300 m; and most of the gullies have a width <10 m, accounting for 50.71% of the total (Table 8). The MBGR of the gully system in the study area is relatively low, with an average MBGR of 0.715, indicating that many gullies in the study area are still in the early stage of development and that the scale of gully erosion is still expanding rapidly. This finding may be due to the overall steepness of the study area, which has an average slope of 32.81° and is conducive to gully development and erosion. The morphological parameters of gully erosion at the same activity level vary greatly and are closely related to the shape of the small watershed, water conditions, land use types, etc. Moreover, 60.56% of the gullies in the study area have a height difference ≥50 m; moreover, the surface cutting depth is relatively high, indicating that the terrain in the study area is highly undulating and that the erosion potential energy of gullies is relatively large, which is conducive to headcut erosion and undercutting erosion processes. However, there are also harsh geological conditions that limit the lateral expansion of gully width. Moreover, the VC rate in the study area is relatively low, and surface runoff easily occurs at relatively high flow velocities and strong erosion forces, resulting in more severe gully erosion.

**Table 8:** Topographic index values of gullies with different activity levels.

Index	Data type	Active	Semiactive	Stable
**Area**	Average	13945.18	12457.15	2491.78
	Maximum	63936.70	29690.53	16297.78
	Minimum	95.09	160.4	12.05
**Perimeter**	Average	1460.56	1231.32	340.93
	Maximum	4846.74	2598.61	2099.01
	Minimum	88.49	107.39	16.01
**Total length**	Average	1272.18	861.33	314.97
	Maximum	7317.25	3140.18	3139.17
	Minimum	37.74	50.4	6.73
**Height difference**	Average	160.34	148.71	49.63
	Maximum	390.52	307.75	223.76
	Minimum	11.21	29.96	1.54
**Average width**	Average	10.31	16.37	6.75
	Maximum	41.77	44.88	44.46
	Minimum	0.94	3.18	0.75
**Surface cutting depth**	Average	0.95	0.98	0.73
	Maximum	1.22	1.13	1.11
	Minimum	0.52	0.78	0.52

### Analysis of factors affecting the gully erosion activity

The formation of gully is influenced by many factors. Previous studies have shown that topographic conditions and land use types are the main influencing factors. The patterns of gully erosion under different conditions, the impact mechanisms, and the dominant driving factors vary geographically, with existing studies mainly focusing on the Yellow River Basin in the Loess Plateau region and the Northeast Black Soil Region, achieving significant results. However, for steep slopes and fragmented terrains with high gradients in the Southwest Mountains, the mechanisms influencing the activity of gullies under unique topography conditions are still unclear. Therefore, we select topography conditions (As, TR, SR and SC) as influencing factors to investigate. Secondly, through field investigations, it was found that the activity of gullies in different land use types within the study area varies significantly. Therefore, we also select the types of land use as one of the influencing factors.

**Fig 7:**
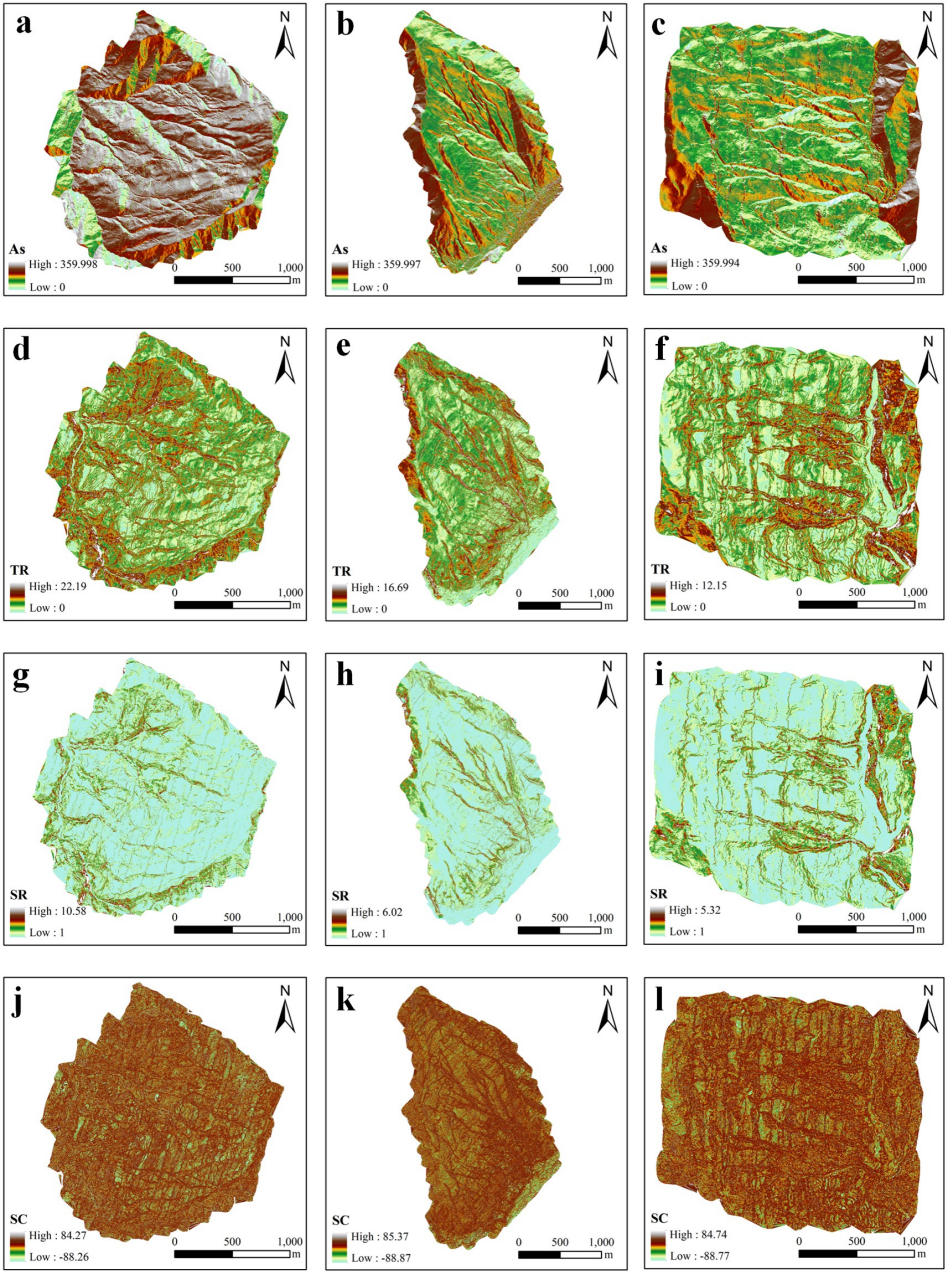
Map of each influence factor. (a-c) The aspect map, (d-f) The topographic relief map, (g-i) The surface roughness map, (j-l) The surface curvature map.

Therefore, this study explored the influence of LUT and topographic conditions on the gully erosion activity and conducted a correlation analysis between the gully activity index and five sets of data: As, SC, TR, SR, and LUT. The results of the Pearson correlation test are shown in Fig 7. There were significant correlations between TR(r = 0.64, *P*<0.01), LUT(r = -0.5, *P*<0.01), SC(r = 0.51, *P*<0.01) and the gully activity index. SR(r = 0.2, *P*<0.01) was positively correlated with gully activity; however, the relationship was not significant. There was no significant correlation between As and gully activity.

**Fig 8:**
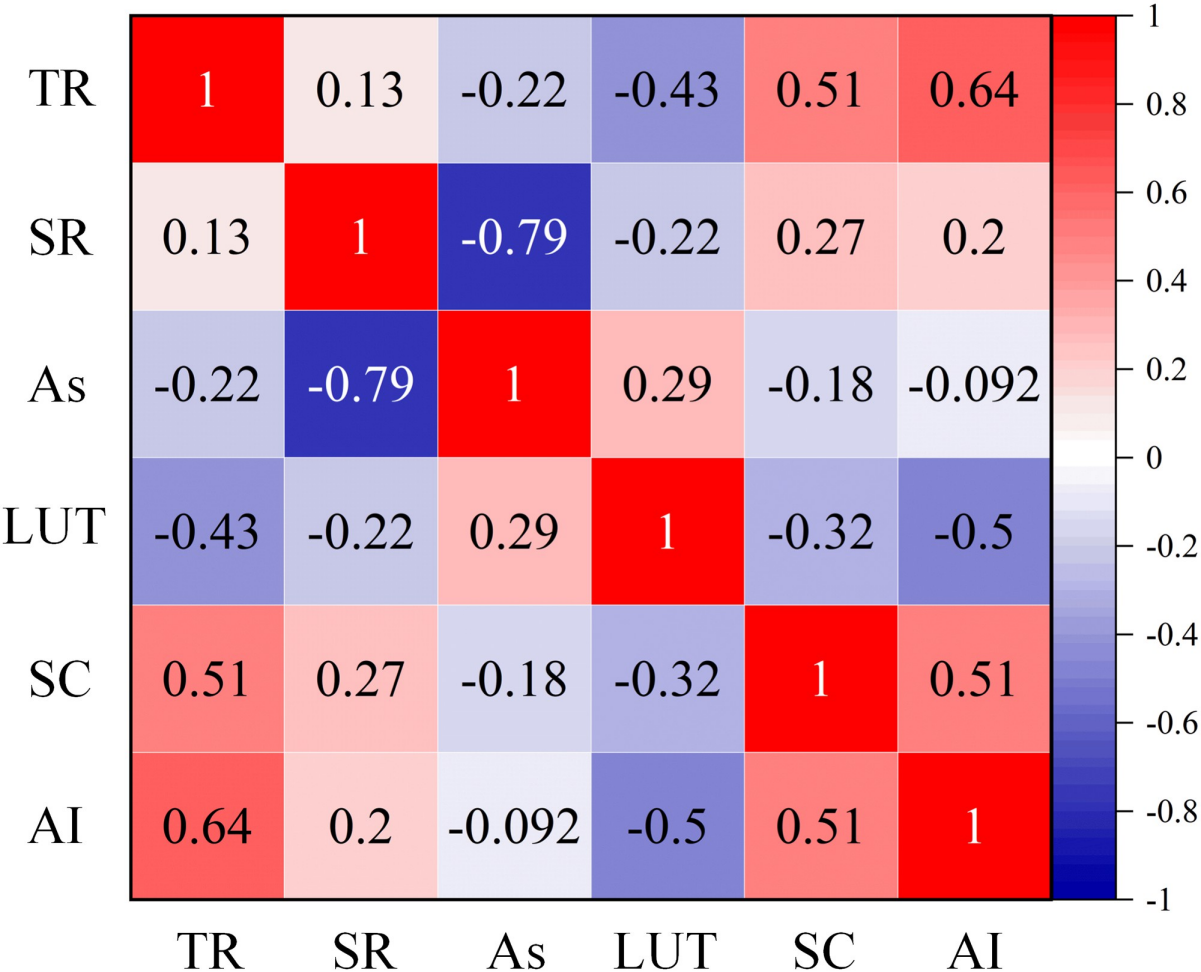
The correlation between gully activity and influence factors. TR represents topographic relief, SR represents surface roughness, As represents slope aspect, LUT represents land use type, SC represents surface curvature and AI represents activity index.

**Fig 9:**
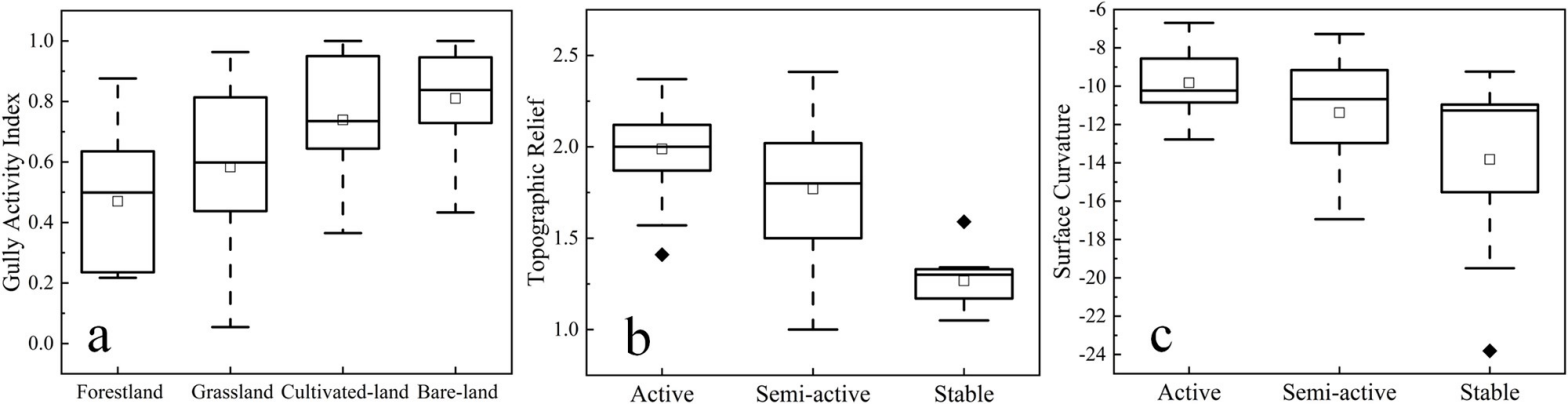
Distribution characteristics of gully activity index on LUTs, TR and SC.

The LUT, TR and SC of the gully erosion in the study area were explored (Fig 8). The average activity indices of the different LUTs in the study area were calculated as follows: forestland (Ai = 0.469), cultivated land (Ai = 0.739), grassland (Ai = 0.583), and bare land (Ai = 0.81) (Fig 9a). The lowest gully activity index was in forestland, and the highest gully activity index was in bare land. The TR of the 71 gullies in the study area ranged from 1.002 to 2.412 (Fig 9b), with average TR of 1.988, 1.769, and 1.263 for active, semiactive, and stable gullies, respectively. The SC ranged from -23.8 to -6.7 (Fig 9c), with active gullies having a SC of -9.83, semiactive gullies having a SC of -11.39, and stable gullies having a SC of -22.78. The SC of the gullies in the study area were all less than 0, indicating that the gullies all had concave slopes.

## Discussion

### Evaluation of research methods

This study innovatively applied the fuzzy mathematical comprehensive evaluation method to identify the activity level of gullies, verifying that this method can be effectively applied to identify the activity level of gullies. Existing research hotspots in soil erosion mainly focus on using machine learning algorithms to identify erosion gullies, assess sensitivity, predict erosion, or identify the main triggering factors in the formation process of gullies [64–66]. However, there is a lack of quantitative expression methods for the specific activity of existing gullies. In areas where direct observation data is lacking, the accuracy of gully erosion estimation models is difficult to effectively verify, and their applicability is also questionable. Indirect methods for model evaluation play a significant role in such areas. The identification of gully activity is influenced by multiple factors, such as topography, meteorological conditions, VC, and soil properties [28]. These factors interact in complex and ambiguous ways. The fuzzy comprehensive evaluation method can quantify this ambiguity and uncertainty [3], making evaluation results more scientific and reasonable. This method considers not only single factors but also their interactions, leading to a more comprehensive evaluation and can distinguish between the gullies that are more active and the gullies that are more stable, which is particularly important for gully activity identification. Moreover, the fuzzy comprehensive evaluation method can select appropriate evaluation indicators and weights based on regional conditions such as geography, climate, and vegetation, making evaluation results more realistic with strong flexibility and adaptability. However, this method also has certain limitations, namely the subjective nature of the division of evaluation index thresholds, which often relies on empirical judgments on the spot, which may lead to differences in evaluation results among different experts on the same issue.

**Fig 10:**
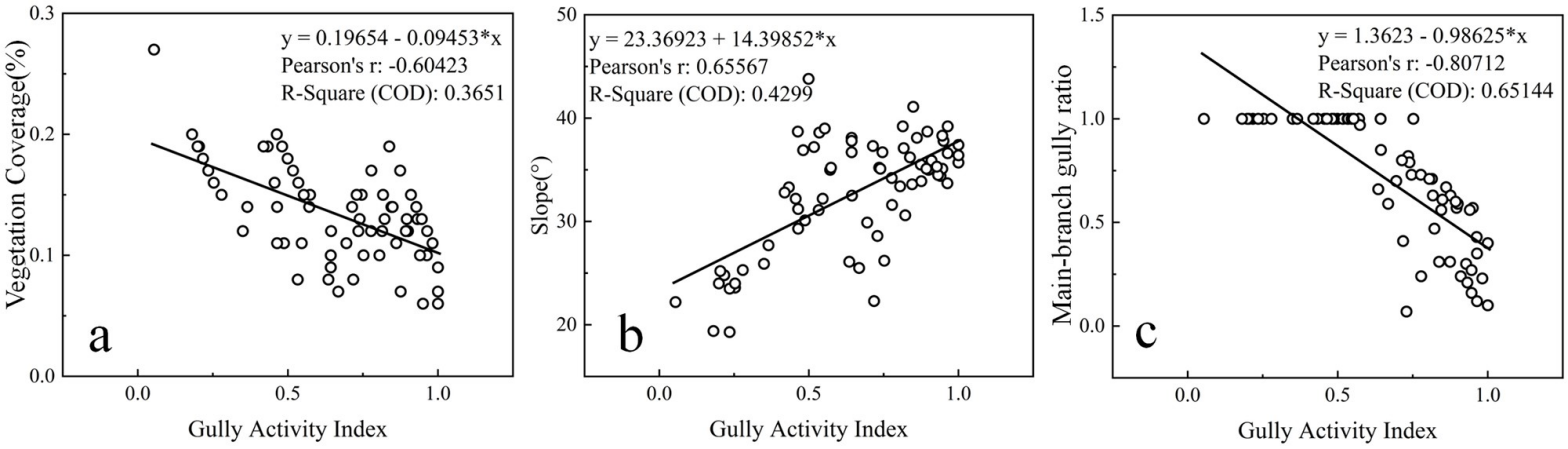
The relationship between activity index and VC (a), slope (b), and MBGR (c).

Therefore, this study used coverage, slope, and the MBGR as the basis, combined with the definition criteria for soil erosion intensity and the actual situation in the study area, the VC, slope, and MBGR are divided into three levels: active (C ≤ 10%, S ≥ 35, MBGR ≤ 0.5), semiactive (10% < C < 30%, 25 < S < 35, 0.5 < MBGR < 1), and stable (C ≥ 30%, S ≤ 25, MBGR ≥ 1). The least significant difference (LSD) one-way ANOVA of VC, slope, MBGR and gully activity index revealed that there were extremely significant differences in VC (F = 14.292, *P*<0.01), slope (F = 34.408, *P*<0.01) and MBGR (F = 44.719, *P*<0.01) among the different gully activity levels (Table 9). This is consistent with existing research findings [67, 68]. Moreover, Pearson correlation analysis was carried out for VC, slope, MBGR and gully activity index. The results revealed that there was a significant negative correlation between gully activity and VC (r = -0.604, *P*<0.01; Fig 10a) and between gully activity and the MBGR (r = -0.807, *P*<0.01; Fig 10c). With increasing VC and MBGR, the gully activity index exhibited a decreasing trend. However, there was a significant positive correlation between gully activity and slope (r = 0.656, *P*<0.01; Fig 10b). With increasing slope, the gully activity index shows an increasing trend. Therefore, VC, slope and MBGR are important factors affecting the gully erosion activity level and are key indicators for characterizing the active gully index in the Sunshui River Basin. As classification indicators, they can be used to classify gully erosion activity effectively and quantitatively.

**Table 9:** Descriptive data and difference test of activity of each evaluation factor.

	Classification of different activity levels			*F*	*P*	Postevent comparison
	C1(n = 31)	C2(n = 31)	C3(n = 9)			
**VC (%)**	0.119±0.031	0.134±0.037	0.188±0.039	14.292	<0.01	C1<C2<C3
**Slope (°)**	35.38±3.03	33.14±5.06	22.89±2.18	34.408	<0.01	C1>C2>C3
**MBGR**	0.45±0.227	0.89±0.213	0.95±0.219	44.719	<0.01	C1>C2>C3

*P*<0.01 indicates extreme significance, 0.01<*P*<0.05 indicates significance, and *P*>0.05 indicates no significance.

### Factors affecting gully activity

LUTs significantly affect the development of gully erosion by influencing runoff processes in catchment areas of gully erosion [69]. The LUTs in the Sunshui River Basin exhibited a significant negative correlation with the gully activity index (r = -0.5, *P*<0.01). When the LUT is bare land (Ai = 0.81), the gullies are the most active, followed by cultivated land (Ai = 0.739) and grassland (Ai = 0.583); however, when the LUT is forestland (Ai = 0.469), the gullies are more stable. Cultivated land is frequently disturbed by human factors, and bare land is exposed due to the lack of vegetation in large areas, facilitating the formation of runoff on slope surfaces; therefore, under these two LUTs, gully erosion is severe. Vegetation leaves in forests and grasslands have a certain interception effect on precipitation, and vegetation roots can increase soil shear strength. The roots and stems of these plants can prevent surface runoff from being collected to some extent [70]. Therefore, more factors slow gully development under these two LUTs, leading to lower gully activity levels. It is similar to the existing research results [71]. There was a close spatial correlation between cultivated land abandonment and severe gully erosion, and due to human activities, natural forestland and grassland were transformed into bare land. When continuous rainfall occurs, discharge greatly increases, leading to faster gully-bank expansion [72]. Therefore, LUTs have a significant impact on gully erosion development, and a correct understanding of land use patterns can help accurately classify the levels of gully erosion activity.

The TR reflects the relative topography of a specific area, and the greater the TR is, the more severe the surface erosion [73]. When studying the TR and geographical significance of the Qinghai–Tibet Plateau, schoolars proposed that the TR is positively correlated with the average altitude and relative height difference [74]. This is consistent with our conclusion. Therefore, in regions with greater terrain fluctuations, the average altitude and relative height difference are greater, and the VC is lower, resulting in a greater surface exposure rate, greater erosion force from precipitation, more severe gully erosion activities, and greater activity levels. There was a significant positive correlation between TR and the gully activity index in the Sunshui River Basin (r = 0.64, P<0.01). The TR of 71 gullies in the region ranged from 1.002 to 2.412, and the average TR of active, semiactive, and stable gullies were 1.988, 1.769, and 1.263, respectively. As gully activity increased, soil erosion became more severe. Therefore, gully activity increased with increasing TR.

The SC is the curved shape of the slope and can indicate the slope type, namely, a concave slope, straight slope, or convex slope. A SC value less than 0 represents a concave slope, and a slope greater than 0 represents a convex slope. If the SC is equal to 0, the slope type is a straight slope [75]. In general, a concave slope has a steeper upper part and a gentler lower part, which is conducive to collecting more runoff on the slope, leading to severe and more active gully erosion. The convex slope is the opposite, with a gentler upper part and a steeper lower part. This slope type is not conducive to the collection of runoff on the slope, and the runoff also consumes some energy to counteract the surface resistance. The erosive force of the runoff is also smaller. A straight slope has roughly the same slope from top to bottom without significant changes, and water usually does not collect or disperse [76]. There was a significant positive correlation between SC and the gully activity index in the Sunshui River Basin (r = 0.51, P<0.01). The greater the SC is, the greater the gully activity level. The 71 gullies in the study area all had concave slopes. Therefore, there is more active gully erosion in the region and less stable gully erosion, which is consistent with the classification results (31 active gullies, 31 semiactive gullies, and 9 stable gullies).

### Work limitations and future outlook

In terms of data processing, the data source of this study is based on UAV aerial photography system. Although UAVs have great advantages in data acquisition, they are greatly affected by environmental conditions during data collection, leading to certain noise effects in data source acquisition and processing. Future research should combine different data acquisition environments and processing methods to expand data volume and improve classification accuracy. In terms of sample size, this study only identified gully activity from topographical conditions, vegetation coverage, and gully morphology. However, gully activity is affected by many factors. Future research should combine meteorological conditions, lithologic character and soil property data to provide a more comprehensive and accurate assessment of gullies. This study accurately identified the specific activity level of gullies in relevant areas and should further improve mitigation measures related to gully erosion to implement different governance methods. For stable gullies with high vegetation coverage, the focus is on maintaining the status quo, supplemented by planting local species to increase species diversity and reduce soil erosion. For semiactive gullies with low vegetation coverage, sowing grass seeds in the gully is implemented, while grass and shrubs are planted at the source to reduce runoff speed and volume entering the gully. For active gullies, prevention and control measures for semiactive gullies are implemented, and embankments are built section by section to reduce sediment transport and prevent further erosion.

## Conclusion

This study investigated the Sunshui River Basin and extracted data on 71 gullies in the study area, including area, perimeter, total gully length, height difference, average width, MBGR, slope, surface cutting depth, and VC data. Field measurements, UAV photography measurements, and remote sensing image extraction methods were used. The gully activity indices (0–0.25 for stable gullies, 0.25–0.75 for semiactive gullies, and 0.75–1 for active gullies) were calculated using the fuzzy comprehensive evaluation method to quantitatively evaluate the gully erosion activity level and elaborate the impact of various factors on gully activity. The validity of the classification method was verified using single factor analysis of variance. The results showed that the activity indices of the 71 gullies ranged from 0.054 to 0.999, with an average value of 0.656. There were 31 active gullies, 31 semiactive gullies, and 9 stable gullies. A total of 87.32% of the gullies in the study area were in the early or middle stage of gully development, with severe gully erosion and soil erosion. Gully activity is affected by multiple factors. The Pearson correlation test results revealed significant correlations between the GAI and topographic relief (r = 0.64, *P*<0.01), land use type (r = -0.5, *P*<0.01), and SC (r = 0.51, *P*<0.01). When the absolute value of surface curvature is large, TR is small, human interference in land use is more frequent, and gully activity is greater; when the absolute value of SC is small, TR is large, and human interference in land use is less frequent, gullies tend to be stable gullies. The quantitative evaluation of gully activity provides new ideas for studying gully development processes; furthermore, this study provides a basis for targeted soil and water conservation measures and a reference for research on related regions and geomorphological information.

## Supporting information

S1 Dataset (ZIP)

S2 Dataset (ZIP)

S3 Dataset (ZIP)

S4 Dataset (ZIP)

S5 Dataset (XLSX)
